# Thyroid Grafting in Human Beings

**Published:** 1904-09-03

**Authors:** 


### THYROID GRAFTING IN HUMAN BEINGS.

The discovery of the functions of the thyroid gland, and the recognition that certain symptoms were associated with deficient development or atrophy of this organ, not unnaturally led to the hope that it might be possible to engraft portions of living thyroid substance with successful results. Experience, however, showed that such grafts, whether taken from animals or human beings, did not long retain their vitality, or exercise a beneficial effect on the patient. In spite of the repeated failures of a number of experimenters in different countries, Dr. H. Christiani[1] is still of opinion that thyroid grafting is a procedure which may one day become a recognised therapeutic measure. Not only is it possible now, he says, to obtain grafts which will endure, but the failures of the past can be explained. The following are the rules which he lays down: (1) the graft must be homo-thyroid; (2) only normal living tissue must be transplanted; (3) small multiple grafts should be made, taking care to implant them in very vascular regions. During an operation upon the neck of a girl, whose thyroid was normal, a graft was taken from the gland and implanted in the supra-clavicular fossa. Six months later the graft was removed, and microscopical examination showed it to have the same structure as the normal gland. The alveoli were large within proper limits, and were filled with colloid substance. In order to have a simpler method, he proposes to use thyroid-gland substance very finely divided with a sharp knife and suspended in serum, the dose being administered hypodermically by means of a needle.

Whatever further experience may show to be possible with regard to treating thyroid deficiency by transplanting healthy thyroid tissue into affected individuals, there will always be the problem of how to obtain the normal tissue so long as it is not possible to use grafts from the lower animals.

[1] Medical Press, Aug. 17.

